# The art of research cohort construction using administrative datasets

**DOI:** 10.23889/ijpds.v9i5.2886

**Published:** 2024-09-18

**Authors:** Lee Williamson

**Affiliations:** 1 University of Edinburgh

## Objectives

Using research case studies, I will give an overview as to how researchers can experience problems translating great research ideas, grounded in the relevant literature, into a robust research design. Assuming that the question cannot be reliably researched using small but rich sample surveys, I will present ways in which routine admin data can help in this context, along with the additional challenges posed when creating the correct cohort to address the research question.

## Approach

The examples given are from a study which links together routinely collected administrative data for a representative sample of the population. It includes a wealth of information from census, civil registration data, education data, and with further permission health data can also be linked. The study in question is provided deidentified, and accessed within a data enclave. The size and scope of the study make it an unparalleled resource for analysing a range of socio-economic, demographic and health questions.

## Results

I will demonstrate how despite the large number of study members, these cohorts must be carefully considered in order to research outcomes (events/results) due to inconsistency in the years that different admin data are available centrally in admin systems (health/education data). Examples include: life-course events for a cohort of women followed up to mid-life, and a complete sub-cohort followed up into later-life.

## Discussion

To showcase exciting ways in which linked admin data can be operationalised to take a longitudinal/life-course approach to address research questions, and to emphasise cohort construction is complex.

